# Parasitization by the wasp *Eretmocerus mundus *induces transcription of genes related to immune response and symbiotic bacteria proliferation in the whitefly *Bemisia tabaci*

**DOI:** 10.1186/1471-2164-9-342

**Published:** 2008-07-18

**Authors:** Assaf Mahadav, Dan Gerling, Yuval Gottlieb, Henryk Czosnek, Murad Ghanim

**Affiliations:** 1 The Hebrew University of Jerusalem, Faculty of Agricultural, Food and Environmental Quality Sciences, The Robert H. Smith Institute of Plant Sciences and Genetics in Agriculture and the Minerva Otto Warburg Center for Agricultural Biotechnology, Rehovot, 76100, Israel; 2 Department of Zoology, Tel Aviv University, Tel Aviv, Israel; 3 Institute of Plant Protection, Department of Entomology, Agricultural Research Organization, The Volcani Center, Bet Dagan 50250, Israel

## Abstract

**Background:**

The whitefly *Bemisia tabaci *(Gennadius) (Hemiptera: Aleyrodidae), and the viruses it transmits, are a major constraint to growing vegetable crops worldwide. Although the whitefly is often controlled using chemical pesticides, biological control agents constitute an important component in integrated pest management programs, especially in protected agriculture. One of these agents is the wasp *Eretmocerus mundus *(Mercet) (Hymenoptera: Aphelinidae)*. E. mundus *lays its egg on the leaf underneath the second-third instar nymph of *B. tabaci*. First instars of the wasp hatch and penetrate the whitefly nymphs. Initiation of parasitization induces the host to form a capsule composed of epidermal cells around the parasitoid. The physiological and molecular processes underlying *B. tabaci-E. mundus *interactions have never been investigated.

**Results:**

We used a cDNA microarray containing 6,000 expressed sequence tags (ESTs) from the whitefly genome to study the parasitoid-whitefly interaction. We compared RNA samples collected at two time points of the parasitization process: when the parasitoid first instar starts the penetration process and once it has fully penetrated the host. The results clearly indicated that genes known to be part of the defense pathways described in other insects are also involved in the response of *B. tabaci *to parasitization by *E. mundus*. Some of these responses included repression of a serine protease inhibitor (*serpin*) and induction of a melanization cascade. A second set of genes that responded strongly to parasitization were bacterial, encoded by whitefly symbionts. Quantitative real-time PCR and FISH analyses showed that proliferation of *Rickettsia*, a facultative secondary symbiont, is strongly induced upon initiation of the parasitization process, a result that supported previous reports suggesting that endosymbionts might be involved in the insect host's resistance to various environmental stresses.

**Conclusion:**

This is the first study to examine the transcriptional response of a hemipteran insect to attack by a biological control agent (hymenopterous parasitoid), using a new genomic approach developed for this insect pest. The defense response in *B. tabaci *involves genes related to the immune response as described in model organisms such as *Drosophila melanogaster*. Moreover, endosymbionts of *B. tabaci *appear to play a role in the response to parasitization, as supported by previously published results from aphids.

## Background

The whitefly *Bemisia tabaci *is one of the most destructive pests to agricultural crops worldwide [1,2]. It vectors many plant viruses [3], feeds on phloem sap, and excretes honeydew that promotes the growth of damaging fungi [1,4]. The whitefly colonizes more than 600 different species of plants in fields and greenhouses and causes yearly losses estimated at billions of dollars [5,6]. While insecticides are often used to control this pest [7], biological control agents, especially various *Eretmocerus *(parasitoid) species, are being mass-reared and released in vegetable crops throughout the world, including the United States and Europe, to assist in the control [8,9]. *Eretmocerus *species exhibit a unique form of immature development. Eggs are laid on the leaf underneath the host nymph; first instars hatch and then penetrate the host [10]. Upon initiation of parasitoid penetration, host epidermal cells and possibly other immune system-derived cells are stimulated to undergo mitosis, forming a capsule around the parasitoid larva which, in contrast to the other known parasitoid-induced capsules, is of epidermal origin [11]. The capsule completely isolates the larva from the host tissues and although its function is not known, it may serve as a nutritional mediator between the host and parasitoid while preventing direct contact between the latter and the host's immune system [10]. Previous studies with various *Eretmocerus *species have shown that all whitefly nymphs except for crawlers and fourth-instar nymphs in the pharate adult stage are susceptible to oviposition by the wasp. However, host penetration occurs only once the whitefly has reached the fourth nymphal stage [12,13]. It is believed that chemical intervention by the hatching parasitoid larva in the developmental processes induces host epidermal cell proliferation and, ultimately, capsule formation [10]. The host response to parasitization has not been investigated at the molecular level. However, a study of the associated hormonal interactions has shown that ecdysteroid levels do not rise and may even decrease in response to parasitization of the pre-penetrated and penetrated fourth nymphal stage [11].

The well-studied system of *Drosophila melanogaster *and its parasitoid wasp *Asobara tabida *(Hymenoptera: Braconidae) may serve as a baseline for a molecular study of the present system, although the biology of the two systems is distinct. Unlike the *B. tabaci-E. mundus *system, *A. tabida *lays eggs inside *Drosophila *larvae rather than underneath them, and the eggs adhere to the internal organs of the host [14,15]. This foreign invasion and direct contact between the host and the parasitoid triggers a process of encapsulation when blood cells (hemocytes) recognize and aggregate around the parasitoid egg. Additional hemocytes then follow suit, resulting in the formation of a multilayer capsule. Thereafter, melanin is deposited on the capsule, killing the parasitoid by asphyxiation or via necrotizing compounds [16,17].

Microarray-based genome-wide analyses have been instrumental in the analysis of *Drosophila*'s immune response upon infection with bacteria, virus and fungi [18-20] and upon parasitization by *A. tabida *[17]. These types of attack induce the expression of common defense pathways which include the endopeptidase *corin*- and *Stubble*-like genes and *easter*- and *snake*-like genes, probably secreted in the hemolymph, genes such as *serpins *and *serine-type endopeptidases *that may be involved in a proteolytic cascade, and humoral defense-related genes of the Toll- and immune deficiency (Imd)-signaling pathways. Other important pathways include the Janus kinase (JAK)/signal transducer and activator of transcription (STAT) as well as cellular defense pathways that lead to phagocytosis and nodule formation. Aside from the genes expressed in response to both bacterial and parasitoid infection, parasitization induces sets of genes that are not activated by bacterial or fungal attack [17].

In this study, we investigated the response of *B. tabaci *to parasitization by the wasp *E. mundus *using a whitefly cDNA-based microarray. We found that parasitization induces regulation of host gene transcription. Some of the targeted genes are related to the immune response and are also regulated in other systems, such as *Drosophila *paratisized by *A. tabida*. We also found that genes of the whitefly's endosymbiotic bacteria respond strongly to parasitization. This result supports previous reports of the involvement of symbiotic bacteria in the resistance of some aphid lines to parasitoids [21].

## Results and discussion

### Microarray hybridization results

**Figure 1:**
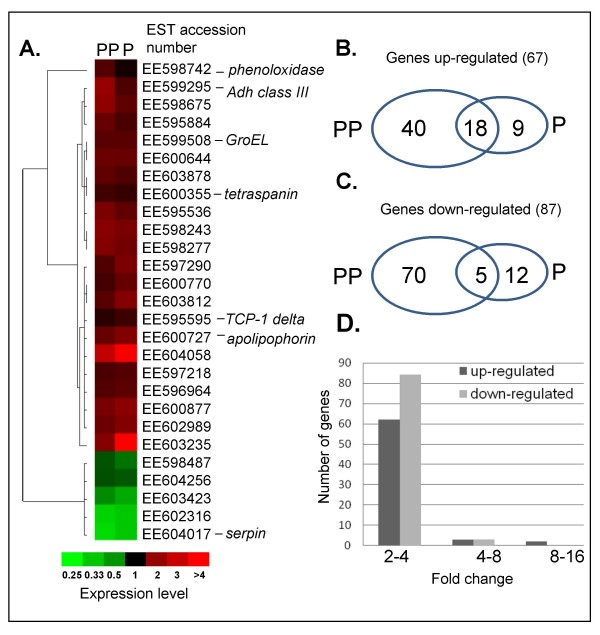
**General statistics on the differentially regulated genes in response to parasitization**. (A) Cluster image of the 23 differentially regulated genes identified by microarray hybridization during both parasitization stages plus identified immune-responsive genes that were followed in further experiments. The expression profiles after pre-parasitization (PP) and full parasitization (P) are shown. Columns correspond to the two time points and rows to the different genes. Red indicates increased mRNA levels, whereas green indicates decreased levels compared with non-parasitized pupae. The brightest reds and greens are sixfold induced and repressed, respectively. The graphs in B and C show the number of genes induced (B) and repressed (C) in response to pre- and full penetration, and the number of genes that showed shared significant expression during both stages. (D) Distribution of induced (black bars) and repressed (gray bars) genes based on their fold change.

Whitefly cDNA-based microarray was used to identify genes expressed as a result of *E. mundus *parasitization of *B. tabaci *at two time points of the parasitization: 1) when the parasitoid first instars are pre-penetrating (PP) *B. tabaci*'s fourth nymphal stage and 2) when they have fully penetrated (P). Parasitized and non-parasitized whitefly nymphs of the same age were compared. Overall, 67 genes (44% of the total number of differentially regulated genes) were differentially up-regulated, of which 18 genes were shared between the two parasitized stages, and 87 genes (56% of the total number of differentially regulated genes) were differentially down-regulated, of which only 5 genes were shared between the two parasitized stages (Figure 1B &1C). Combining these data, 23 genes showed significant changes over the two time points tested and were shared between the two parasitized stages. Aside from these 23 genes, others did not necessarily show any increase over time, i.e. as parasitization progressed. The expression patterns of the 23 genes that showed differential changes in both parasitized stages and of an additional four immune-responsive genes that were further investigated are shown in Figure 1A. Plotting the distribution of the regulated genes based on their fold change showed that the expression of most of them (more than 95%) changed between two- and fourfold, while only a few of the genes changed more than fourfold (Figure 1D). See additional files 1 and 2 for a complete list of the 67 up-regulated and 87 down-regulated genes, respectively. These files present data regarding best hits in GenBank, annotation of the differentially regulated sequences, E-values, Gene Ontology and other useful information (where available).

### Modulation of expression of whitefly stress and immune-response genes in response to parasitization

The response to several biotic stress factors (bacteria, fungi, parasitoids, viruses) and injury has been studied in model insects such as *D. melanogaster *and *Anopheles gambiae *[17,18], [22,23]. Gene expression of *Drosophila *upon parasitization by *A. tabida *revealed a set of unique responsive genes, as well as genes responding to other biotic stresses [17]. Hence, following microarray hybridization, we screened our database [24] and identified several genes with homologues known to be involved in stress responses in other insects, such as *serpin A3K *(serine protease inhibitor), *phenoloxidase*, *tetraspanin D107*, *apolipophorin *and *alcohol dehydrogenase class III *(*adhIII*).

**Figure 2:**
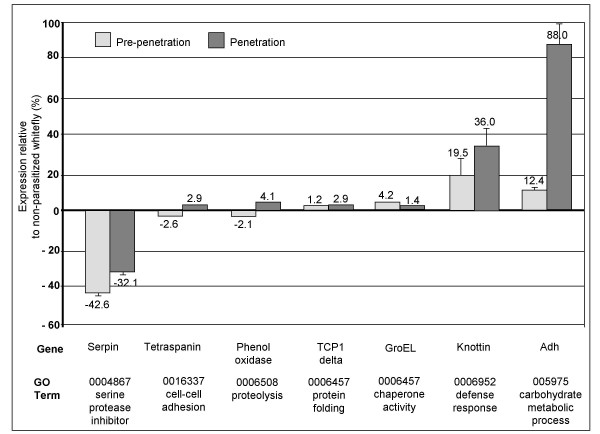
**Quantitative real-time RT-PCR verification of candidate genes induced or repressed following the microarray analyses**. Further description is provided in the manuscript. For each gene verified, the available Gene Ontology (GO) term is given underneath the gene name.

*Serpin A3K *was one of the genes showing the highest level of down-regulation as a result of parasitization at both the PP and P stages: microarray analyses indicated 5.9-fold (PP) and 6.5-fold (P) repression. Quantitative RT-PCR analyses (Figure 2) confirmed the strong repression of *serpin *(by 42.6- and 32.1-fold at the PP and P stages, respectively). Serine proteases and their inhibitors (serpins) have been shown in several invertebrates to be involved in an early response to stress and in the activation of defense mechanisms, including hemolymph coagulation [25], melanization [26] and induction of antibacterial peptides [27]. The results suggest that in *B. tabaci*, serpins act as inhibitors of the cascade leading to melanization. Indeed, parasitization induces down-regulation of *serpin *and initiation of the melanization cascade characterized in some insects by pigmentation and wound-healing.

Moreover, a *phenoloxidase *gene was found to be induced by approximately fourfold in microarray analyses (Figure 1). Quantitative RT-PCR analysis showed that this gene is repressed at the PP stage but is induced by over fourfold at the P stage (Figure 2), confirming that parasitization had activated the melanization molecular cascade. The down-regulation of *phenoloxidase *at the PP stage may further support the fact that the melanization cascade is molecularly activated, and since *phenoloxidase *is a final step in this cascade, its up-regulation is observed only when the parasitoid has fully penetrated the host during the P stage. Up-regulation of *phenoloxidase *genes in response to biotic stresses such as parasitoid attack in *Drosophila *[17] and to the immunosuppressive polydnavirus HdIV in *Spodoptera frugiperda *[28] has been previously documented. Phenoloxidase has also been shown to catalyze the conversion of dopamine to melanin, which is toxic to microorganisms [29]. Although we did not observe any melanin around the parasitoid penetration area, and the spatial expression of the phenoloxidase gene following whitefly nymph parasitization is not known, the molecular events following the parasitization process support activation of the phenoloxidase cascade in parasitized *B. tabaci*. The incomplete epidermal layer of cells formed around the parasitoid larva has been suggested to protect it from the host's immune response by compartmentalizing it and creating an environment that is suitable for the provision of nutritional substances during the penetration process [10]. The host response to parasitization may give some clue as to the evolution of defense mechanisms in insects, and the ways in which parasitoids overcome these mechanisms. *E. mundus *lays its egg outside the host body and is therefore considered an exoparasitoid. The parasitoid penetrates the host's body later on, as a first instar that chews into the whitefly nymph from the outside. This parasitization process may be sufficient to activate the molecular cascade, but final melanization is not observed. This incomplete process may enable *E. mundus *to avoid the whitefly nymph's defense response. We concluded that whereas endoparasitism in the *Drosophila-A. tabida *system leads to full melanization, in the *B. tabaci-E. mundus *system, the parasitoid overcomes the host's defense system by initializing the parasitization process outside the host, thus avoiding the insect's defense response.

Three *knottin *sequences have been previously shown to be abundant in cDNA libraries prepared from whiteflies carrying begomoviruses [30]. An increase in the expression of these knottins was observed after the acquisition of begomoviruses by whiteflies, supporting previous studies that showed a decrease in whitefly fertility, fecundity and longevity after begomovirus retention in the insect body [31]. Knottins are miniproteins that are present in many diverse organisms and possess various biological functions [32]. For instance, knottins have toxic properties in plants, bugs, mollusks and arachnids, and are antimicrobial agents in plants, insects and arthropods. Although knottin peptide sequences were not represented on our microarray, we manually identified a *knottin *sequence that had been deposited in GenBank (Table 1) [30]. qRT-PCR showed a high level of induction of this sequence, reaching 19.5- and 36-fold at the PP and P stages, respectively. This result could be related to serpin's down-regulation. Indeed, serpins are also known to inhibit the Imd- and Toll-signaling pathways, which lead to the production of antimicrobial peptides in the fat body cells [18]. Therefore *serpin *down-regulation may play a role in the induction of defense molecules during parasitoid invasion.

**Table 1:** Oligonucleotide primers used in quantitative PCR and quantitative RT-PCR. Amplicon sizes for all genes are 81 bp.

Gene	EST accession number	Primer sequence (5' to 3')
*actin*	EE597333	TGGAGATGGTGTTTCCCACAC
		CCAGCCAAGTCCAAACGAAG
*serpin*	EE604017	GCTCGACCATGGACTGGTTC
		CTAGATTTCGCCGCGGTAGT
*phenoloxidase*	EE598742	GGCGAGGAGAAGGACTGTGA
		AATAAGGCAGACCCCATCGG
*tetraspanin-3*	EE600355	GCATCGGTCAGATCGTGTTG
		CTTTCAAGGAGCCGAAGCAT
*Adh-classIII*	EE599295	GGTTATGTCTGCTCCTGCCG
		CCCAAAAGTTCAGCAGCCTC
*TCP1-delta*	EE595595	CGGCTTCAAATAGTTCAGGTGA
		TTCGAATATCCTTTGGCTTGCT
*GroEL*	EE599508	GTTGTAGCTGGAGGAGGTACTGACC
		TGTTTGGTCTTCGTTGTTGCC
*citrate synthase gltA*	DQ077708	AAAGGTTGCTCATCATGCGTT
		GCCATAGGATGCGAAGAGCT
*knottin*	DQ308607	CTGTTCCAAGCCAAAACCGA
		GATCATGAAGGCGGCCACTA

Among the defense-response genes discovered in our study was *tetraspanin D107*. Tetraspanins are transmembrane proteins that are involved in a wide variety of fundamental biological processes [33,34] and modulate signal transduction by interacting with (immuno)receptors and signaling molecules. Some tetraspanin genes, such as *tetraspanin CD37*, are expressed exclusively in the immune system [35]. It has been suggested that their expression changes during infection [36] and that these molecules play an important role in the immune response to pathogens. In our microarray screen, the expression of *tetraspanin D107 *was induced twofold upon parasitization in the P stage but did not show any significant change during the PP stage. qRT-PCR indicated this gene's down-regulation (2.6-fold) at the PP stage and up-regulation (2.9-fold) at the P stage (Figure 2). It is not unusual for the qRT-PCR approach to identify significant changes in gene expression that are not identified in the larger microarray screen, as we observed with *tetraspanin D107 *in the PP stage. The fact that *tetraspanin D107 *was not significantly up-regulated at the PP stage supports the hypothesis that this protein is involved in a final step of the response to parasitoid attack.

Other induced immune system-related genes included the *T-complex protein 1 delta subunit *(*TCP1-delta*) involved in defense and homeostatic responses [37] (Figure 2), and *apolipophorin*. Among other functions, apolipophorin has been identified as an immune-activating protein. This protein, which is abundant in the insect hemolymph, is believed to cause hemagglutination [38] and to act synergistically with the hemolymph lysozyme [39]. Elevated apolipophorin titers have been observed during programmed cell death of intersegmental muscle cells [40], detoxification [41], and induction of antimicrobial peptides [42]. The *apolipophorin *that we identified was up-regulated by 3.5-fold at the PP stage and by 2.5-fold at the P stage. Although we did not confirm this expression pattern by qRT-PCR, we believe that this gene's response to parasitization is part of the general immune response to parasitoid invasion.

An interesting finding from our screen was the strong induction of an *alcohol dehydrogenase III (adhIII) *gene during both stages of parasitization: more than two- and fourfold at the P and PP stages, respectively. Interestingly, these results were confirmed by qRT-PCR, but the induction was much higher than that observed in the microarray experiment, reaching an over 12-fold increase at the PP stage and an approximately 88-fold increase at the P stage (Figure 2). These high rates of induction are remarkable and indicate an important role for this gene in response to parasitoid invasion. The ADH locus and its genes (*adh*) have been much studied by population and evolutionary biologists, but their functions have not been fully elucidated [43]. One study found a significant decrease in ADH activity and accumulation under conditions of heat stress in *Drosophila*; however, this protein's involvement in stress resistance was never fully addressed [44]. Our results suggest a strong involvement of the *adh class III *gene in the response to parasitization and further experiments are required to determine whether this is a general stress response or one that is specific to parasitoid invasion.

### Response of whitefly endosymbionts to parasitization

**Figure 3:**
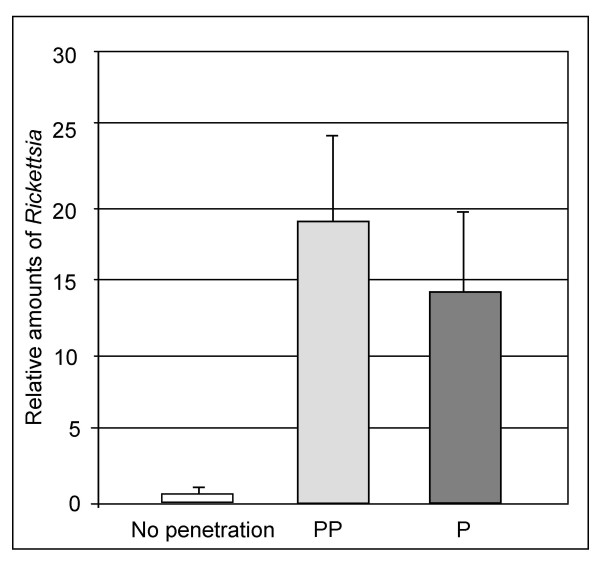
**Quantitative real-time PCR of *Rickettsia***. Control non-parasitized (No penetration) *B. tabaci *instars (PP) compared to pre-penetrated and penetrated (P) instars by *E. mundus*.

**Figure 4:**
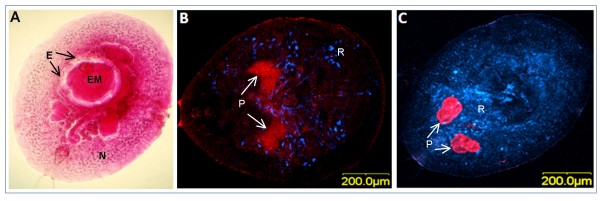
**Fluorescent *in-situ *hybridization (FISH) of parasitized *B. tabaci *pupae by *E. mundus***. (A) An *E. mundus *second-instar larva (EM) within a fourth-stage nymph of *B. tabaci *(N). Note that the parasitoid larva is surrounded by a cellular capsule (E) enclosing a translucent region, thus preventing direct contact with the host. (B) FISH of non-parasitized *B. tabaci *pupa with the primary symbiont *Portiera *(P) labeled with Cy3 (red) and *Rickettsia *(R) labeled with Cy5 (blue). (C) FISH of parasitized *B. tabaci *pupa by *E. mundus *with *Portiera *(P) and *Rickettsia *(R) labeled as in B (after the wasp larva was dissected out). Note the higher concentration of labeled *Rickettsia *cells in the parasitized pupa compared to the non-parasitized one.

Several genes associated with bacterial genomes rather than *B. tabaci *itself were discovered in our microarray experiments. *B. tabaci *hosts the obligatory bacterium *Portiera aleyrodidarum*, which supplements the whitefly's imbalanced sap diet. In addition, *B. tabaci *populations may harbor a diverse array of different facultative bacterial tenants, including *Hamiltonella*, *Arsenophonus*, *Wolbachia*, and *Rickettsia *[45]. These facultative secondary symbionts may benefit host fitness under specific environmental conditions (heat stress, available host plant, natural enemies) or manipulate the reproduction of their hosts in ways that enhance their own transmission (inducing parthenogenesis, feminizing genetic males, male-killing, cytoplasmic incompatibility) [46-48]. Our present study showed that many significantly regulated genes, belonging to several bacterial metabolic pathways, were associated with the primary symbiont *Portiera *and the facultative symbiont *Rickettsia*. Most noticeable was a *Portiera GroEL *homologue, a chaperone involved in protein folding and protein-protein interactions, which was induced by over threefold at the PP stage, but not at the P stage. This result was verified by qRT-PCR analysis (Figure 2). Since the role of the primary symbiont *Portiera *in *B. tabaci *is defined and resembles the function of other primary symbionts in other arthropods, i.e. supplementation of the whitefly's imbalanced diet, and since it is confined to the bacteriome, a fact that may limit its effect on the response to external stress factors, we decided to focus on the response of the secondary symbiont *Rickettsia. Rickettsia *was the only secondary symbiont found in the whitefly population we studied, and is usually not confined to the bacteriome but rather is scattered throughout *B. tabaci*'s body, reaching almost all of its organs [48,49]. Thus, it is expected that only this bacterium will exhibit a noticeable phenotype if whitefly endosymbionts are involved in the response to parasitization. The amount of *Rickettsia *cells was quantified by qPCR before and during parasitization. The interesting results, summarized in Figure 3, showed an approximately 20-fold increase in the amount of *Rickettsia *as a result of parasitization at the PP stage, and a ca. 15-fold increase at the P stage. This observation was confirmed using a fluorescent *in-situ *hybridization (FISH) approach. Figure 4A shows the ventral side of a *B. tabaci *nymph (N) parasitized and penetrated by an *E. mundus *(EM) first instar, and the epidermal capsule layer formed around the penetration area (E). Figure 4B shows a FISH assay targeting *Portiera *(P, red) and *Rickettsia *(R, blue) in a non-parasitized nymph. Figure 4C shows a parasitized *B. tabaci *nymph penetrated by the *E. mundus *first instar in the early stages of penetration. The fluorescent signal associated with *Rickettsia *in the parasitized nymph is far stronger than in the non-parasitized nymph, unlike the signal from *Portiera *which seems to be unchanged. This result strongly supports our qPCR results, and the hypothesis that *Rickettsia *is involved in the response to parasitization by *E. mundus*. The strong response of *Rickettsia *to wasp invasion can be explained by either bacterium-host or bacterium-invader cross-talk. In the first case, the insect host may mobilize the free *Rickettsia *cells to counter the wasp's development. In the second, the endosymbiont might sense chemicals secreted by the parasitoid or by the parasitized host and induce the latter's response. The immune response observed in the whitefly nymph following parasitization by *E. mundus *is probably not due to the increased concentration of *Rickettsia *because a comparison, using microarrays, of gene expression in whitefly populations containing *Rickettsia *and those without *Rickettsia *revealed no significant regulation of the immune response-related genes identified in this study (M. Ghanim, unpublished).

The involvement of symbiotic bacteria in their insect host's stress response has been shown in aphids, in which parasitoid attack [21,46] and other stress factors, such as high temperatures [50,51] and fungal attack [52], are influenced by secondary symbionts. It has been recently suggested that a toxin produced by the symbiont is involved in killing invading wasps [53]. Although survival of *B. tabaci *nymphs from isofemale lines with and without *Rickettsia *after parasitization by *E. mundus *does not show any significant differences (D. Gerling, unpublished), *Rickettsia *induction following invasion by *E. mundus *may play an important role in these host-parasite interactions, and successful parasitism needs to be compared between whiteflies with and without *Rickettsia*.

## Conclusion

The molecular mechanisms used by hemipterans to combat attacks by biological control agents are completely unknown. We used a recently developed microarray for *B. tabaci *to examine the response of this insect to the unique attack of the biological control wasp *E. mundus*. Although similar research approaches have been published on model insects such as *Drosophila *and mosquitoes with parasitic wasps, no data were available in non-model insects. Over 20 genes responded consistently to the wasp attack at the two time points investigated, suggesting their role in the immune response. Interestingly, some symbiotic bacterial genes also showed a significant response to the wasp attack. We concluded that the facultative secondary symbiont *Rickettsia *may play an important role in the response to attacks by this wasp. qPCR and FISH analyses showed a strong *Rickettsia *response and suggested its direct recognition of the invasion, either by recognizing the invader or by recognizing the whitefly's stress response via secreted molecules. The involvement of symbiotic bacteria in their hosts' stress response is intriguing and further research should focus on the mechanisms by which symbionts sense the stress situation and respond to it.

## Methods

### Insect rearing, whitefly parasitization by *E. mundus *and sample collection for microarrays

*B. tabaci *biotype B were reared on cotton plants in growth chambers [54] at 25 ± 2°C, photoperiod 16:8 L:D. *E. mundus *was maintained on whitefly-infested cotton plants housed in Perspex insect-proof cages with mesh-covered windows. Whiteflies in the second to third stage of nymphal development were used for parasitization, whereas all of the material for the experiments was taken from early fourth-instar nymphs. Two stages of parasitization were compared with developmental stage-matched non-parasitized nymphs. The first stage consisted of pre-penetration (PP) *B. tabaci *nymphs, in which the freely moving mouthparts of the first, newly hatched wasp were still exposed, prior to penetration. The second stage consisted of a penetrating parasitoid larva half to fully embedded in the host with its extremity still protruding (P). The latter was extirpated from the host. In both stages the wasp larva was removed from the host into TRI reagent (MRC Inc., Cincinnati, OH, USA) before nucleic acid extraction to avoid contamination. As controls, 30 to 40 whitefly nymphs at the same stage of development were collected for each replicate. A total of three replicates were used, showing correlations of over 80%.

### RNA isolation, labeling and hybridization

Pre-penetrated and penetrated as well as non-parasitized pupae of *B. tabaci *were collected and homogenized in TRI reagent. The total RNA served as a template for quantitative real-time RT-PCR (qRT-PCR) analyses and for linear amplification [55]. The amplified RNA was reverse transcribed (SuperscriptII kit; Invitrogen, Paisley, UK) and aminoallyl-labeled using the TIGR protocol available online at . Three replicates of non-parasitized pupae were coupled with Cy3-ester and directly hybridized against three replicates of pre-penetrated or fully penetrated instars, which were also coupled with Cy5-ester. Following purification (Qiagen PCR purification kit; Hilden, Germany), the labeled cDNAs were mixed in 3× SSC (supplemented with 20 μg poly(A) and 0.15% SDS) and hybridized to the *B. tabaci *microarray in a 65°C water bath for 16–18 h. *B. tabaci *microarray construction and description were as previously described [54]. The slides were sequentially washed (2 min per wash) at ambient temperature in 1.14× SSC supplemented with 0.0285% SDS, 1.14× SSC, 0.228× SSC and 0.057× SSC. Immediately after washing, arrays were spun dry at 1000 *g *for 5 min in a table-top centrifuge. The slides were scanned using an Agilent microarray scanner (Agilent Technologies, Santa Clara, CA, USA) to detect Cy3 and Cy5 fluorescence. The ratio of the two dyes was used as an indicator of the relative abundance of the two mRNA transcripts. The data generated in this study were deposited in NCBI's Gene Expression Omnibus (GEO, ) and are accessible through GEO series accession number GSE11410.

### Quantitative real-time PCR (qPCR) and qRT-PCR analyses

The expression of genes selected from the results of the microarray hybridizations was verified using a qRT-PCR approach. The genes, accession numbers, amplicon sizes and primers are shown in Table 1. Amplifications were performed using 1× Quantitect SYBR Green PCR mix (Qiagen) and 5 pmol of each primer. *B. tabaci actin *DNA was used as an internal standard for data normalization and quantification. To ensure the validity of the data, the expression of each gene was tested in triplicate in each of three biologically independent experiments. The cycling conditions were: 15 min activation at 95°C, 45 cycles of 10 s at 95°C, 20 s at 60°C, 25 s at 72°C. Melting ramp from 60°C to 99°C, rising by 1°C at each step, and waiting 5 s after each step. Channel source: 470 nm, detector: 510 nm. A Rotor-Gene 6000 machine (Corbett Robotics Pty Ltd., Brisbane, Australia) and the accompanying software were used for qPCR, data normalization and quantification. The *actin *gene was used as internal control. Quantification of *Rickettsia *was performed by qPCR using the citrate synthase gene (*gltA*) [56].

### Microarray data analysis

Fluorescence of the hybridizing spots was collected using the Genepix 4000B scanner. Fluorescence intensity was quantified using the Genepix Pro v6 (Axon

Instruments, Union City, CA, USA). The experiments were performed in triplicate and were reproducible, with Pearson correlation coefficients between repeat experiments for the genes that passed the significance test used (see below) of 0.8. The expression data obtained across the two time points investigated were processed using the Linear Models for Microarray Data analysis package LIMMA [57]. Probes that were not present in all of the arrays were removed from the data before normalization. Data from the arrays were subjected to "normexp" background subtraction, followed by *LOESS *within-array normalization and "Gquantile" between-array normalization. Replicates were merged using median values. To identify the genes whose expression varied the most across each developmental time point, we used multivariate empirical Bayesian modeling as carried out in the time-course package [58]. This modeling takes into account correlations across time points and multiple replicates to derive posterior odds for the differential gene expression and produces a ranked list of genes. We calculated a *P*-value for each gene from its multivariate empirical Bayesian score for differential expression using F-distribution (*k*, *m*+*n*+ν-*k*-*1*), where: *k *= number of time points, *m *and *n *are number of replicates for two compared developmental stages and ν is the prior degree of freedom. Results were considered significant at *P *< 0.05. All hierarchical clustering in our analyses was performed and viewed with Gene Cluster and TreeView softwares [59]. Average linkage hierarchical clustering was used to group genes according to shared expression profiles. Parameters used in the clustering were hierarchical clustering, by genes, using average linkage clustering. The Pearson correlation coefficient distance metric was used in the clustering analysis.

### Florescence *in-situ *hybridization (FISH)

The FISH procedure followed Gottlieb et al. [48], using the probe BTP1-Cy3 (5'-Cy3-TGTCAGTGTCAGCC CAGAAG-3') to detect *Portiera*, and the probe Rb1-Cy5 (5'-Cy5-TCCACGTCGCCGTCTTGC-3') to detect *Rickettsia*. Stained samples were mounted whole and viewed under an IX81 Olympus FluoView500 confocal microscope. Specificity of detection was confirmed using the following controls: no-probe control, RNase-digested control, and *Rickettsia*-free whiteflies.

## Authors' contributions

AM carried out the microarray experiments and the qPCR analyses. DG carried out the parasitization experiments and dissections, and provided the whitefly samples. YG conducted the FISH analyses. MG designed the experiments with HC, DG and AM, performed the statistical analyses and drafted the manuscript. HC and YG helped to finalize the manuscript. The final version of the manuscript was read and approved by all authors.

## Supplementary Material

Additional file 1 Genes significantly up-regulated following parasitization of *B. tabaci *by *E. mundus*. The data consist of 67 significantly up-regulated genes following parasitization of *B. tabaci *by *E. mundus*. The list is divided into three parts: genes that are up-regulated in both the pre-penetration (PP) and penetration (P) stages, genes that are uniquely up-regulated in the PP stage and genes that are uniquely up-regulated in the P stage. The table presents data regarding best hits in GenBank, annotation of the differentially regulated sequences, E-values, Gene Ontology and other useful information (where available). Click here for file

Additional file 2 Genes significantly down-regulated following parasitization of *B. tabaci *by *E. mundus*. The data consist of 87 significantly down-regulated genes following parasitization of *B. tabaci *by *E. mundus*. The list is divided into three parts: genes that are down-regulated in both the pre-penetration (PP) and penetration (P) stages, genes that are uniquely down-regulated in the PP stage and genes that are uniquely down-regulated in the P stage. The table presents data regarding best hits in GenBank, annotation of the differentially regulated sequences, E-values, Gene Ontology and other useful information (where available). Click here for file
